# Targeting Cellular Metabolism With CPI-613 Sensitizes Pancreatic Cancer Cells to Radiation Therapy

**DOI:** 10.1016/j.adro.2022.101122

**Published:** 2022-11-09

**Authors:** Husain Yar Khan, Mandana Kamgar, Amro Aboukameel, Sahar Bannoura, Brian Y. Chung, Yiwei Li, Mohammed Najeeb Al Hallak, Philip A. Philip, Susan Tsai, Sanjeev Luther, William A. Hall, Asfar S. Azmi

**Affiliations:** a Department of Oncology, Karmanos Cancer Institute, Wayne State University School of Medicine, Detroit, Michigan; b Medical College of Wisconsin, Milwaukee, Wisconsin; c Henry Ford Health Systems, Detroit, Michigan; d Cornerstone Pharmaceuticals, Cranbury, New Jersey

## Abstract

**Purpose:**

Local tumor progression is a cause of significant morbidity and mortality in patients with pancreatic ductal adenocarcinoma (PDAC) with surgically unresectable disease. Novel and effective approaches to accomplish durable local control are urgently needed. We tested whether CPI-613 (devimistat), a first-in-class investigational small molecule inhibitor of mitochondrial metabolism, was capable of altering cancer cell energy metabolism and sensitizing PDAC cells to radiation therapy (RT).

**Methods and Materials:**

The effect of a combined treatment of RT with CPI-613 on the viability of, clonogenic potential of, and cell death induction in PDAC cells (MiaPaCa-2 and Panc-1) was determined using a trypan blue dye exclusion assay, a colony formation assay, and a 7–amino-actinomycin D assay, respectively. The synergistic effects of CPI-613-RT and chemotherapeutic agents (gemcitabine or 5-fluorouracil) were measured in MiaPaCa-2 cells using a 3-(4,5-dimethylthiazol-2-yl)-2,5-diphenyltetrazolium bromide and spheroid formation assay. Changes in energy metabolism were determined by profiling metabolites treated with either RT, CPI-613, or both using liquid chromatography-mass spectrometry.

**Results:**

This study demonstrates that a combination of single-fraction RT (2 and 10 Gy) with CPI-613 significantly inhibits PDAC cell growth compared with RT alone. Molecular analysis revealed inhibition of α-ketoglutarate dehydrogenase at the protein level. In addition, we demonstrate enhanced cell death of PDAC cells when treated with RT-CPI-613 combination. Targeted metabolomic analysis on PDAC cells post–CPI-613-RT treatment revealed alterations in key mitochondrial metabolites, with broader target engagement by the combination treatment, indicating the sensitization of CPI-613–treated PDAC cells to RT. Furthermore, a combination treatment of CPI-613 with either gemcitabine or 5-fluorouracil in the presence of 2 Gy RT synergistically inhibits PDAC cell proliferation.

**Conclusions:**

Our results support a novel combination of CPI-613-RT that warrants further preclinical and early-phase clinical investigations. A phase 1 trial designed to identify the maximum tolerated dose of CPI-613 in combination with chemo-RT in patients with PDAC was recently initiated (NCT05325281).

## Introduction

Pancreatic cancer remains an aggressive and lethal disease and is projected to be the second leading cause of death due to cancer in the United States by 2025.^1^ Local tumor progression is the leading cause of hospitalization for those with unresectable pancreatic ductal adenocarcinoma (PDAC), a substantial contributor to pancreatic cancer morbidity and responsible for mortality in one-third of patients.^2^, ^3^, ^4^ Although conventional chemoradiotherapy among patients with locally advanced PDAC modestly improves local disease control,^5^ the risk of local progression remains significant. Recently, ablative radiation has shown impressive results in select patients with PDAC.^6^ However, the use of ablative radiation is limited to those patients with anatomically favorable tumors.^7^^,^^8^ An urgent need exists to develop innovative therapeutic strategies that effectively control local PDAC progression.

It is well-known that metabolic reprogramming and enhanced mitochondrial function, both hallmarks of PDAC biology, facilitate tumor cell plasticity and promote chemo- and radio-resistance.^9^ Cancer cell mitochondria are critical regulators of deranged tumor metabolism and have been shown to guide molecular pathways involved in radio-resistance.^10^ Emerging data also indicate that mitochondrial metabolites modulate the response of cancer cells to radiation therapy (RT) primarily by affecting the ability to repair DNA.^11^ These observations make them optimal candidates for novel radio-sensitization strategies, as these characteristics are unique to PDAC cells and are limited in normal cells.

CPI-613, a lipoic acid analog, selectively inhibits pyruvate dehydrogenase and α-ketoglutarate dehydrogenase/2-oxoglutarate dehydrogenase (OGDH) in tumor cells, resulting in disruption of the Krebs cycle and ultimately leading to selective tumor cell death.^12^ CPI-613 in combination with standard-of-care chemotherapies has shown significant antitumor activity and low toxicity in patients with metastatic PDAC.^13^^,^^14^ We hypothesized that administration of CPI-613 in combination with conventional RT, either with gemcitabine (Gem-RT) or without (RT), would induce a metabolic shift away from mitochondrial respiration, thereby enhancing tumor cell death compared with conventional therapies alone.

## Methods and Materials

### Cell lines, drugs, and irradiation

MiaPaCa-2 and Panc-1 cells were purchased from American Type Culture Collection (ATCC, Manassas, VA). MiaPaCa-2 and Panc-1 cell lines were maintained in Dulbecco's modified Eagle medium (Thermo Fisher Scientific, Waltham, MA) supplemented with 10% fetal bovine serum, 100 U/mL penicillin, and 100 μg/mL streptomycin in a 5% CO_2_ atmosphere at 37°C. Cell lines were tested and authenticated by the Applied Genomics Technology Center, a core facility, by short tandem repeat profiling using the PowerPlex 16 System (Promega, Madison, WI). Mycoplasma testing was routinely performed on cell lines by polymerase chain reaction. All experiments were performed within 20 passages of the cell lines. CPI-613 (Adooq Bioscience, Irvine, CA) was dissolved in dimethyl sulfoxide to make 200 mM stock solutions. The drug control used for in vitro inhibitor experiments was cell culture media containing 0.1% dimethyl sulfoxide. Radiation treatment was done using an X-RAD 320 Irradiator (manufactured by Precision X-Ray) using the following settings: 320 kV; 12.5 mA; dose = 319.1 cGy/min (filter: 2 mm Al, source to specimen distance: 50) 2 Gy-38 seconds and 10 Gy-188 seconds.

### Trypan blue assay

Cells were seeded at a density of 5 × 10^4^ viable cells/well in 6-well plates (Costar, Cambridge, MA) and were subjected to treatment with either CPI-613, radiation, or both the next day. After 72 hours of treatment, cell viability was determined by a trypan blue dye exclusion test (trypan blue [0.4%]; Sigma Chemical Co, St Louis, MO).

### Preparation of total protein lysates and Western Blot analysis

Cells (1 × 10^6^ MiaPaCa-2) were grown in 100-mm Petri dishes overnight. The following day, cells were treated with either CPI-613 alone, radiation alone, or both for 24 hours. Cells were lysed in radioimmunoprecipitation assay buffer to extract total protein, and protein concentrations were measured using a bicinchoninic acid protein assay (PIERCE, Rockford, IL). Protein lysates (40 μg total) from treated and untreated cells were resolved using 10% sodium dodecyl-sulfate polyacrylamide gel electrophoresis and transferred onto a nitrocellulose membrane. Membranes were incubated with the following primary antibodies (Cell Signaling Technology, Danvers, MA) at 1:1000 dilution in 3% nonfat dry milk: anti-OGDH (#26865) and anti–pyruvate dehydrogenase (#3205). Anti-β-actin (#sc-47778; Santa Cruz Biotechnology, Santa Cruz, CA) was used at a dilution of 1:3000. Incubation with 1:2000 diluted horseradish peroxidase-linked secondary antibodies (#7074/7076; Cell Signaling, Danvers, MA) in 3% nonfat dry milk was subsequently performed at room temperature for 1 hour. The signal was detected using the ECL chemiluminescence detection system (Thermo Fisher Scientific).

### Colony formation assay

MiaPaCa-2 and Panc-1 cells were seeded at a density of 50,000 cells per well in 6-well culture plates and treated with either CPI-613 alone, radiation alone, or a combination of both for 72 hours. After treatment, cells were trypsinized and 500 cells were replated in 6-well plates for an additional 10 days. After the incubation period, media was removed from the wells, and colonies were fixed with methanol and stained with crystal violet for 15 minutes. The plates were then washed and air dried before colonies were photographed.

### Quantification of nonviable cells by 7-amino-actinomycin D

Viable, nonviable, and apoptotic cells were detected using a 7-amino-actinomycin D (7-AAD) assay (Calbio-chem-Novabiochem, La Jolla, CA) and flow cytometric analysis. PDAC cells (MiaPaCa-2 and Panc-1) were seeded in 6-well culture plates at a density of 50,000 viable cells per well and exposed to either CPI-613 alone, radiation alone, or a combination of both for 72 hours. After treatment, cells were collected, washed with phosphate-buffered saline (PBS), and stained with 7-AAD. Stained cells were then analyzed using an FACScan (Becton Dickinson, Mountain View, CA). Data on 20,000 cells were acquired and processed using the Lysys II software (Becton Dickinson). Scattergrams were generated by combining forward light scatter with 7-AAD fluorescence.

### MTT assay and synergy analysis

MiaPaCa-2 cells were seeded in 96-well culture plates at a density of 3 × 10^3^ cells per well and treated the next day with either CPI-613 alone, Gem/5-fluorouracil (5-FU) alone, or in combination with one another, followed by exposure to 2 Gy radiation. After 72 hours, an MTT (3-[4,5-dimethylthiazol-2-yl]-2,5-diphenyltetrazolium bromide) assay was performed according to procedures described previously.^15^ The resulting cell proliferation data (6 replicates per treatment) were used to perform synergy analysis employing the CalcuSyn software (Biosoft, Cambridge, UK).

### Spheroid formation assay

MiaPaCa-2 cells were trypsinized, collected as a single-cell suspension using a 0.45 μm cell strainer, and resuspended in 3-dimensional (3D) tumorsphere medium XF (PromoCell, Heidelberg, Germany). One thousand cells were plated in each well of an ultralow attachment 6-well plate (Corning, Durham, NC). Cells grown in a spheroid formation medium were treated with either CPI-613 alone, Gem alone, or in combination, followed by exposure to a 2 Gy dose of radiation (3 replicates for each treatment). After treatment, spheroids were observed under an inverted microscope and images were captured at 10 × magnification.

### Metabolomic analysis

Cells (1 × 10^6^ MiaPaCa-2) were seeded in 60-mm Petri dishes and incubated at 37°C in a 5% CO_2_ incubator until the cells were 80% confluent. Cells were then either irradiated using a 2 Gy dose of radiation, exposed to 200 μM of CPI-613, or a combination of both for 6 and 24 hours. Each treatment was carried out in 5 replicates. After the treatment period, cells were washed twice with ice-cold PBS, the PBS was completely removed, and cells were collected in 1 mL ice-cold methanol. Changes in metabolites were detected by liquid chromatograph-mass spectrometry at the Karmanos Pharmacology Core.

### Statistical analysis

Wherever appropriate, the data were subjected to a Student *t* test using GraphPad Prism software (La Jolla, CA). *P* < .05 was considered statistically significant.

## Results

### RT in combination with CPI-613 reduces the viability of PDAC cells in vitro and enhances CPI-613-induced downregulation of α-ketoglutarate dehydrogenase

**Figure 1:**
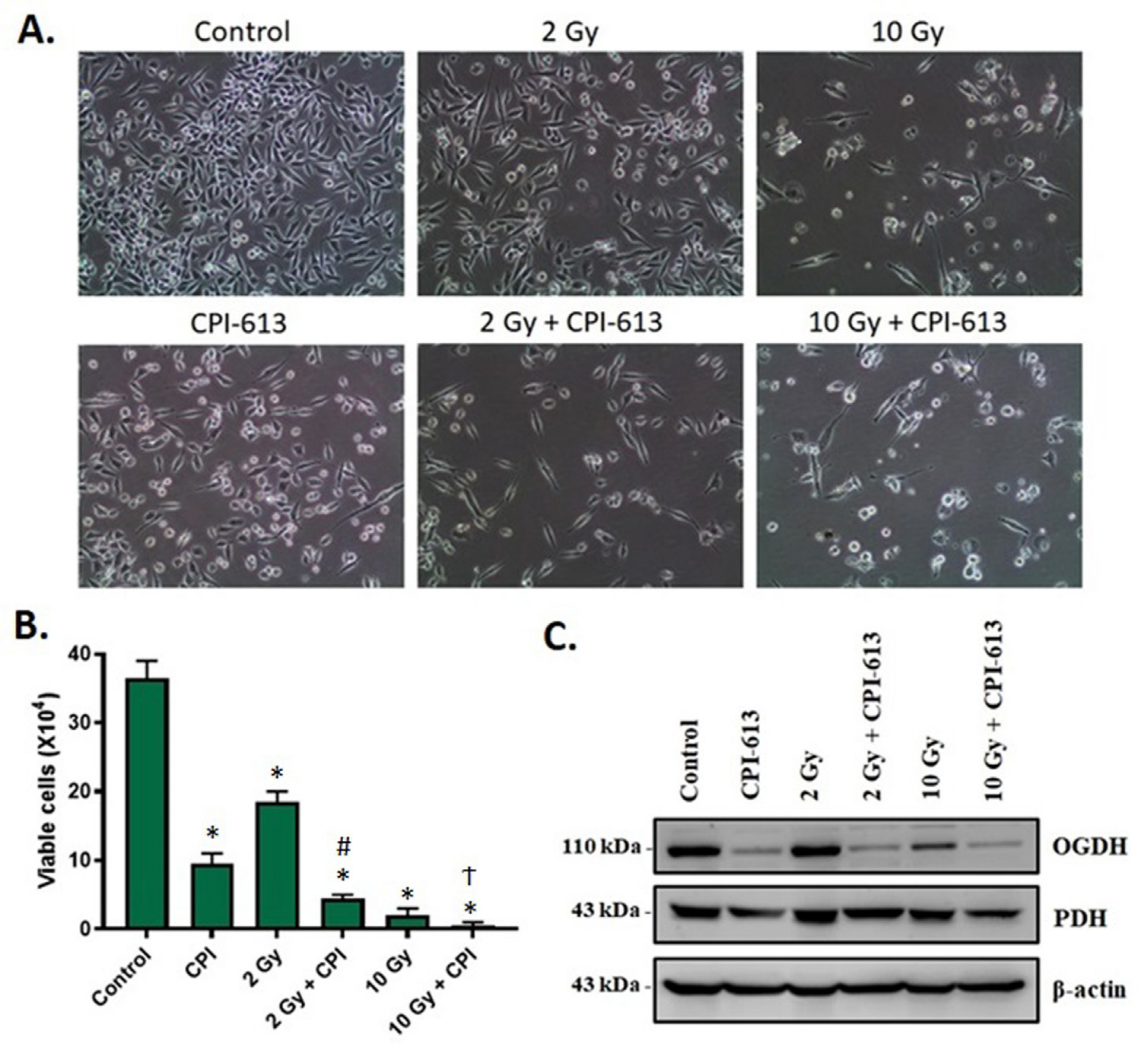
Effect of combining radiation therapy (RT) with CPI-613 on the viability of pancreatic ductal adenocarcinoma (PDAC) cells and the cellular targets of CPI-613. (A) Representative photomicrographs of cells subjected to the indicated doses of radiation and 300 μM CPI-613 for 72 hours were captured at 10 × magnification on an inverted light microscope. (B) Cell viability was determined by trypan blue dye exclusion assay at the end of the experiment and plotted as average number of viable cells ± standard error of the mean (SEM) of 2 replicates. *P < .05 compared with control. #*P* < .05 to 2 Gy RT alone. Ϯ*P* < .05 compared with CPI-613 alone. (C) Protein expression of pyruvate dehydrogenase (PDH) and 2-oxoglutarate dehydrogenase (OGDH) (cellular targets of CPI-613) in MiaPaCa-2 cells irradiated with 2 or 10 Gy radiation and treated with 300 μM of CPI-613 for 72 hours was analyzed by Western blotting.

RT targets nuclear and mitochondrial DNA and therefore, we anticipated that mitochondria-targeted agents would synergize with RT-based therapies. To validate this, we evaluated the effect of an RT-CPI-613 combination treatment on the viability of PDAC cells in vitro*.* As evident from the photomicrographs of MiaPaCa-2 cells subjected to RT-CPI-613 combination treatments, 2 and 10 Gy doses of RT showed enhanced cytotoxicity when combined with CPI-613 (Fig. 1A). Viability of MiaPaCa-2 cells was significantly inhibited by the combination treatments (Fig. 1B). Similar results were obtained with Panc-1 cells (Fig. E1). Western blot analysis showed pronounced downregulation of the mitochondrial tricarboxylic acid cycle enzyme OGDH in MiaPaCa-2 cells that received RT-CPI-613 combination treatments (Fig. 1C).

### RT and CPI-613 combination suppresses the clonogenic potential of PDAC cells

**Figure 2:**
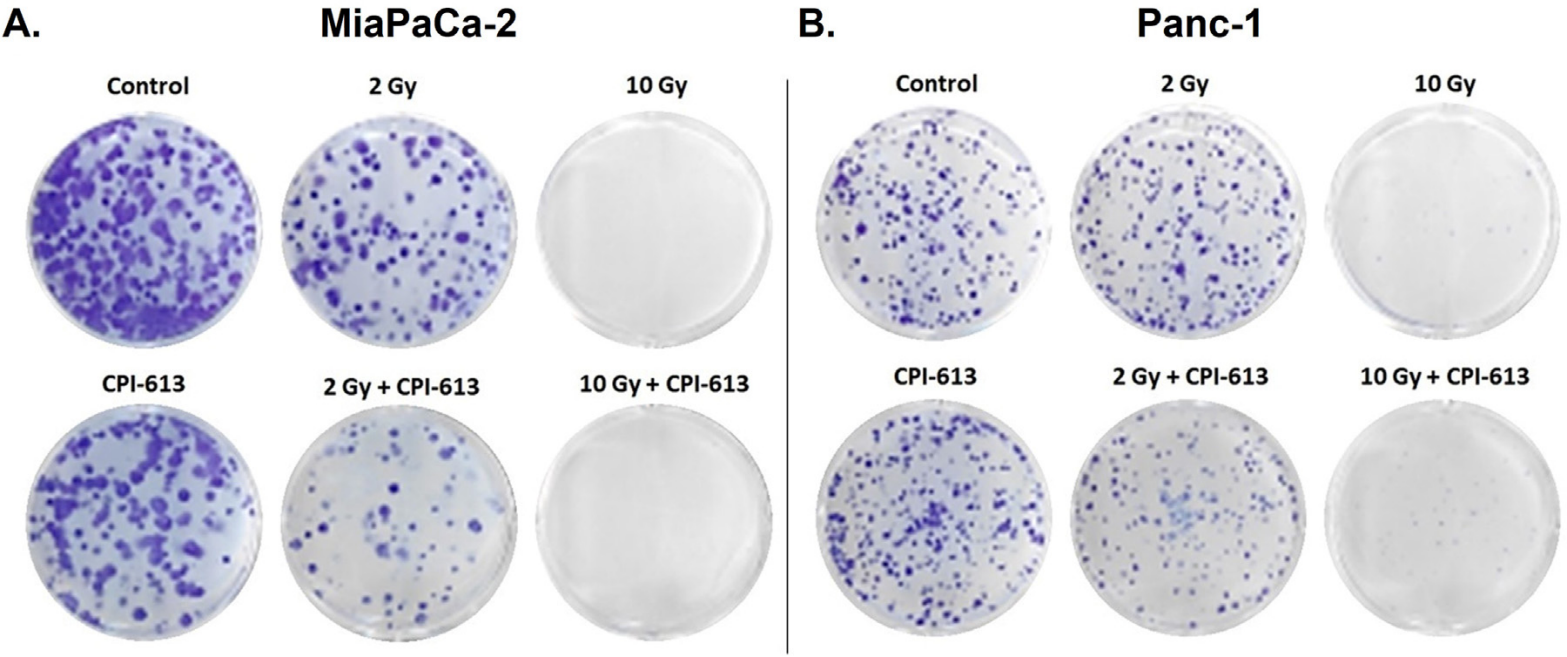
Effect of radiation therapy (RT)–CPI-613 combination on the colony formation potential of pancreatic ductal adenocarcinoma (PDAC) cells. Representative images of colonies formed by (A) MiaPaCa-2 and (B) Panc-1 cells exposed to the indicated doses of radiation either in the absence or presence of CPI-613 (200 μM). Colony formation assay was performed as described in Methods and Materials.

In a colony formation assay, a reduced number of colonies was observed in RT-CPI-613 combination compared with single-agent treatments, indicating that the combination treatment effectively suppressed the ability of MiaPaCa-2 and Panc-1 cells to form colonies. Suppression of colony formation was more pronounced using a higher dose of RT (10 Gy; Fig. 2).

### Combination of CPI-613 with RT enhances PDAC cell death

**Figure 3:**
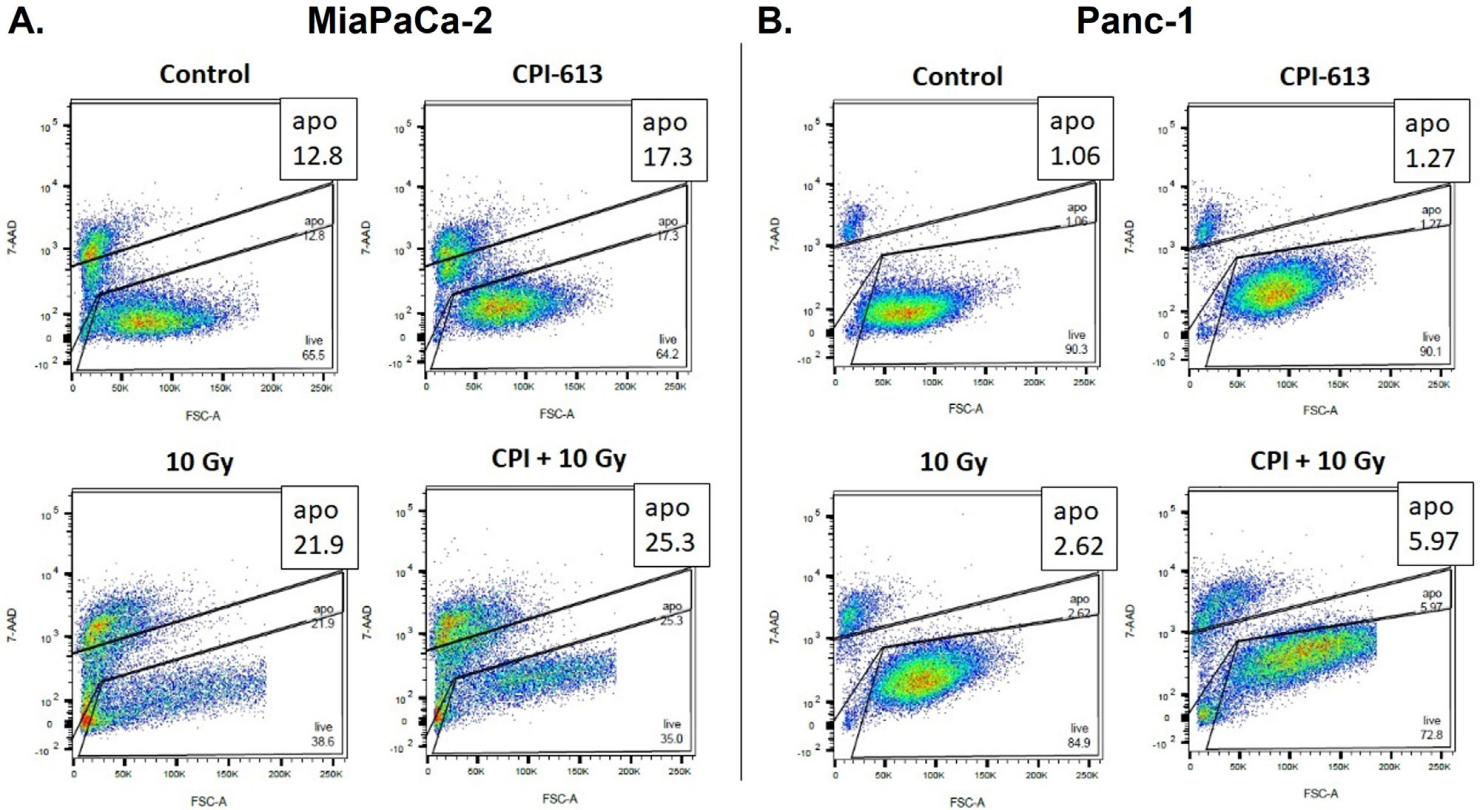
Effect of radiation therapy (RT)–CPI-613 combination on inducing cell death in pancreatic ductal adenocarcinoma (PDAC) cells. Scattergrams showing flow cytometric analysis of apoptotic cells using 7-amino-actinomycin D (7-AAD) assay where MiaPaCa-2 (A) and Panc-1 (B) cells were exposed to 10 Gy radiation either in the absence or presence of CPI-613 (200 μM).

Given that CPI-613 affects mitochondrial enzymes and the mitochondria are the hub for intrinsic apoptosis, we evaluated whether the combination of RT and CPI-613 could induce cell death in PDAC cells. The results shown in Fig. 3 clearly demonstrate that compared with single-agent treatment, the combination effectively enhanced cell death in MiaPaCa-2 and Panc-1 cells, as detected by a 7-AAD assay. These results further corroborate our hypothesis that RT-CPI-613 is an effective combination regimen against difficult-to-treat PDAC.

### CPI-613 synergizes with chemo-RT

**Figure 4:**
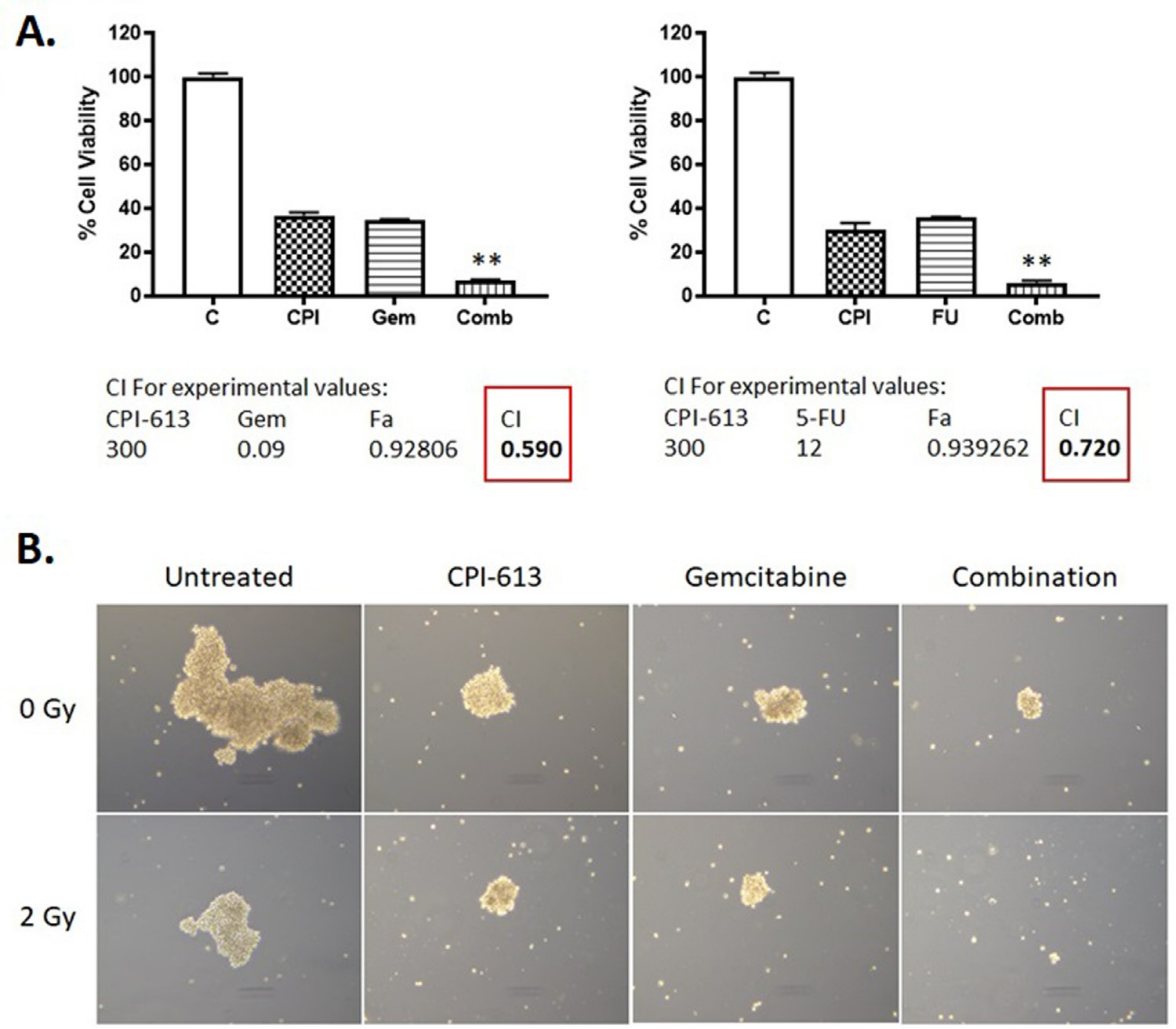
Effect of combining radiation therapy (RT)–CPI-613 with chemotherapeutic drugs. (A) MiaPaCa-2 cells were treated with either gemcitabine (Gem; 90 nM) or 5-fluorouracil (5-FU; 12 μM) in the presence of CPI-613 (300 μM) followed by exposure to 2 Gy RT. A 3-(4,5-dimethylthiazol-2-yl)-2,5-diphenyltetrazolium bromide (MTT) assay was performed after 72 hours of treatment. Cell viability (% of control) was plotted as an average of 6 replicates ± standard error of the mean (SEM). Combination index (CI) was calculated using CalcuSyn software. CI < 1 indicates synergistic effect. **P < .01. (B) MiaPaCa-2 cells were seeded in ultralow attachment plates and later treated with Gem (100 nM) or CPI-613 (300 μM) either as single agents or in combination followed by irradiation with 2 Gy radiation. Each treatment was performed in triplicate. After 1 week, spheroids were observed under an inverted light microscope, and their images were captured at 10 × magnification.

We then tested whether adding Gem or 5-FU to CPI-613-RT further enhanced the efficacy of the combination. As seen in Fig. 4A, the addition of either Gem or 5-FU enhanced the efficacy of CPI-613-RT. Synergy analysis using the CalcuSyn software showed combination index values of less than 1, which indicate synergistic effects of the combination on cell growth inhibition. However, the Gem-CPI-613-RT synergy value was lower than 5-FU-CPI-613-RT. The synergistic effect of combining Gem with CPI-613 was also observed using a 3D spheroid formation assay. Gem-CPI-613 reduced the size of spheroids formed by MiaPaCa-2 cells compared with either of the single agents (Fig. 4B; top row). This effect was further augmented by low-dose RT (Fig. 4B; bottom row), which underscores the efficacy of the Gem-CPI-613-RT combination regimen for PDAC therapy.

### RT-CPI-613 combination treatment alters metabolites in PDAC cells

**Figure 5:**
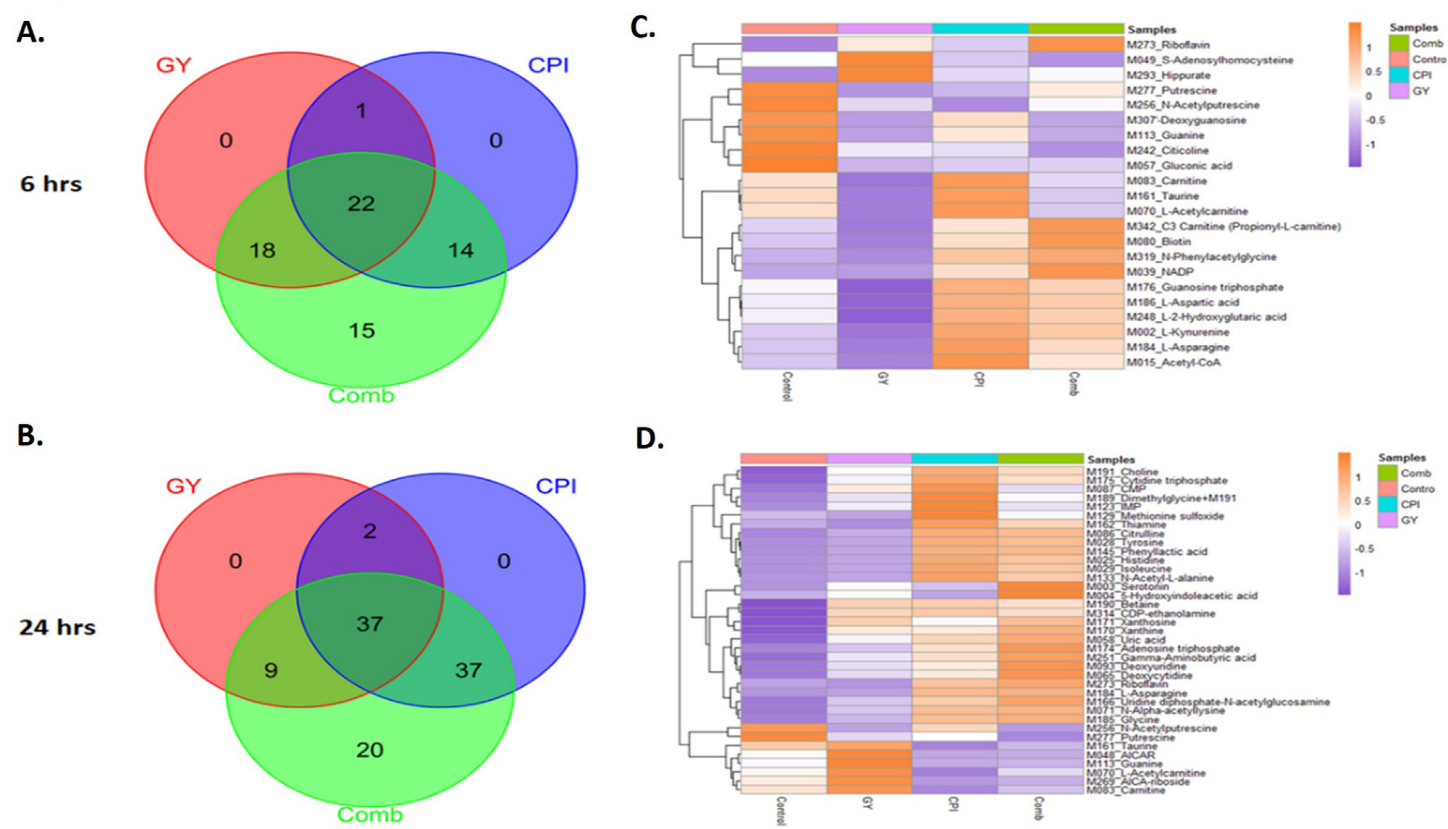
Metabolomic analysis of metabolites altered upon CPI-613–radiation therapy (RT) combination treatment. MiaPaCa-2 cells were exposed to either CPI-613, 2 Gy RT, or their combination for 6 or 24 hours. Targeted metabolomics was then performed as described in Methods and Materials. Statistically significant and differentially expressed metabolites are presented (*P* < .05). (A, B) Venn diagram showing increased number of altered metabolites in the combination. (C, D) Heat maps showing top prioritized targets in 6 and 24 hours of treatment, respectively.

Given the wide-ranging targets altered by RT and several mitochondrial targets of CPI-613, reductionist evaluations of selected pathways would not be an efficient way to understand the synergy between RT and CPI-613. Taking a holistic approach, we performed targeted metabolomic analysis post–CPI-613-RT to understand the spectrum of pathways altered upon combination treatment. As seen in Fig. 5A and 5B, a greater number of metabolites were altered in the combination treatment compared with single-agent treatment. We also observed a unique set of metabolites altered in the combination treatment at 6 and 24 hours (Fig. 5C, 5D). The effect on metabolites was greater at 24 hours compared with 6 hours. A total of 103 metabolites showed significant alterations (*P* < .05) after 24 hours of treatment, while only 69 metabolites were significantly altered after 6 hours treatment. These results demonstrate that RT-CPI-613 combination induces a metabolic change in PDAC cells and further support the use of metabolism-targeting agents in combination with RT for PDAC.

## Discussion

PDAC is a devastating disease that carries an average 5-year survival rate of about 11%, among the lowest of all cancers in the United States.^16^ Although local tumor progression is responsible for mortality in approximately one-third of patients with PDAC,^3^ conventional chemoradiation falls short in providing durable local control of tumor in a significant subset of patients with locally advanced disease.^5^ Here, we provide in vitro evidence demonstrating that CPI-613 may serve as a vital component of a more effective chemo-RT regimen for PDAC treatment. We observed that PDAC cells pre-exposed to a 2 Gy dose of RT and treated with CPI-613 caused a significant decline in cell viability compared with cells receiving RT alone. A similar effect was also seen with a relatively high dose of single fraction RT (10 Gy). Further, using a clonogenic assay, we found that a combination of CPI-613 with a 2 Gy dose of RT leads to a remarkable reduction in the number and size of colonies formed by PDAC cells compared with those formed by cells treated with either therapy alone. Moreover, CPI-613, combined with 5-FU or Gem, exhibited synergistic effects on the growth inhibition of PDAC cells subjected to RT.

Our study, for the first time, reports the potential value of CPI-613 as a radiosensitizer. The safety and efficacy of CPI-613 in combination with chemotherapy have been evaluated previously in early phase PDAC trials.^13^^,^^14^ Given these promising findings, a multicenter phase 3 randomized trial (AVENGER 500) was conducted to evaluate the efficacy of CPI-613 and modified FOLFIRINOX (mFFX) compared with FOLFIRINOX (FFX) in treatment-naïve patients with metastatic PDAC.^17^ Median overall survival (OS), the primary endpoint for the trial, was not statistically significant between the 2 arms. Although the study did not meet the primary endpoint, a post hoc subgroup analysis of female patients with an Eastern Cooperative Oncology Group performance score of 0 revealed CPI-613 and mFFX was associated with a significant increase in median OS (20.4 months, n = 28) compared with FFX (10.4 months, n = 30; hazard ratio, 0.44; *P* = .03).

Although the AVENGER 500 trial failed to demonstrate that CPI-613 in combination with mFFX chemotherapy improves OS in patients with metastatic PDAC, our preclinical studies reported here were designed to evaluate a different indication: the value of CPI-613 as a radiosensitizer. We intend to follow up on these findings by conducting a phase 1 dose-escalation study (NCT05325281) of CPI-613 in combination with chemo-RT in patients with PDAC deemed inoperable (locally advanced, oligometastatic, or medically inoperable) that, by institutional pancreatic tumor board or multidisciplinary review, would otherwise benefit from definitive local control of the primary tumor. Because the efficacy and safety of capecitabine in combination with CPI-613 has yet to be tested and given the preclinical data demonstrating that CPI-613-RT shows greater synergy with Gem than 5-FU (Fig. 4), our trial will use a Gem-based (400 mg/m^2^, once weekly) chemotherapy schedule with RT (Gem-RT). Gem-RT will be administered in combination with long-infusion CPI-613 (starting dose 500 mg/m^2^ with a maximum tested dose of 1500 mg/m^2^) once per week over a 6-week period. Because the post hoc results of AVENGER 500 suggested possible differences in outcomes among female patients, we will also evaluate the efficacy of CPI-613 in combination with Gem-RT based on sex as an exploratory endpoint.

There are some limitations to the preclinical approach and data examining CPI-613’s ability to radiosensitize PDAC cells. First, we used 2 established cell lines to draw our conclusions. Although 3D cultures were also used, the model is derived from cell lines and not primary PDAC tissue-derived cells. The use of Kras, p53, and Cre mouse tumor-derived cells, primary cells, or organoid models would have further strengthened our findings. We also showed the efficacy of a CPI-613-RT combination strategy using exclusively in vitro assays. The combination could be tested in patient-derived or cell line-derived in vivo tumor models. Finally, our metabolomic analysis revealed a number of key synergy metabolites that were not validated using other assays. Additional work may identify actionable targets and biomarkers for the planned clinical study combining CPI-613-RT in metastatic PDAC. Despite these limitations, our findings provide a proof of concept on the utility of CPI-613 in combination with Gem-RT for PDAC. We expect to validate our in vitro findings using xenograft models alongside phase 1b/2 clinical investigations.

## Conclusion

Increasing the effectiveness of conventional chemoradiation therapies using CPI-613 has the potential to improve local control of disease, enhance patient quality of life, decrease morbidity, and extend OS without additional toxicity to the patient. Furthermore, better local control will lead to a decrease in disease-related hospitalization and a reduction in medical care costs related to complications caused by local progression (eg, biliary obstruction, systemic infection due to such obstruction, gastrointestinal bleeding, gastrointestinal obstruction, and pain). The ongoing phase 1 clinical trial will determine the maximum tolerated dose of CPI-613 along with Gem-RT (NCT05325281). We further plan to test the effectiveness of this combination in a multicenter phase 2 study in the neoadjuvant locally advanced setting.

## Acknowledgements

The authors acknowledge the help from Dr Jessica Back of the Microscopy, Imaging and Cytometry Reseources Core in conducting the radiation treatment experiments. The Microscopy, Imaging and Cytometry Resources Core is supported, in part, by NIH Center grants P30 CA22453 to the Karmanos Cancer Institute and R50 CA251068-01 to Dr Moin, Wayne State University, and the Perinatology Research Branch of the National Institutes of Child Health and Development.

## Appendix Supplementary materials

Image, image 1
