# Supplementary figures and images for: Targeting Cellular Metabolism With CPI-613 Sensitizes Pancreatic Cancer Cells to Radiation Therapy
Source: Adv Radiat Oncol. 2022 Nov 9;8(1):101122. doi: 10.1016/j.adro.2022.101122 (PMC9720358; doi:10.1016/j.adro.2022.101122)

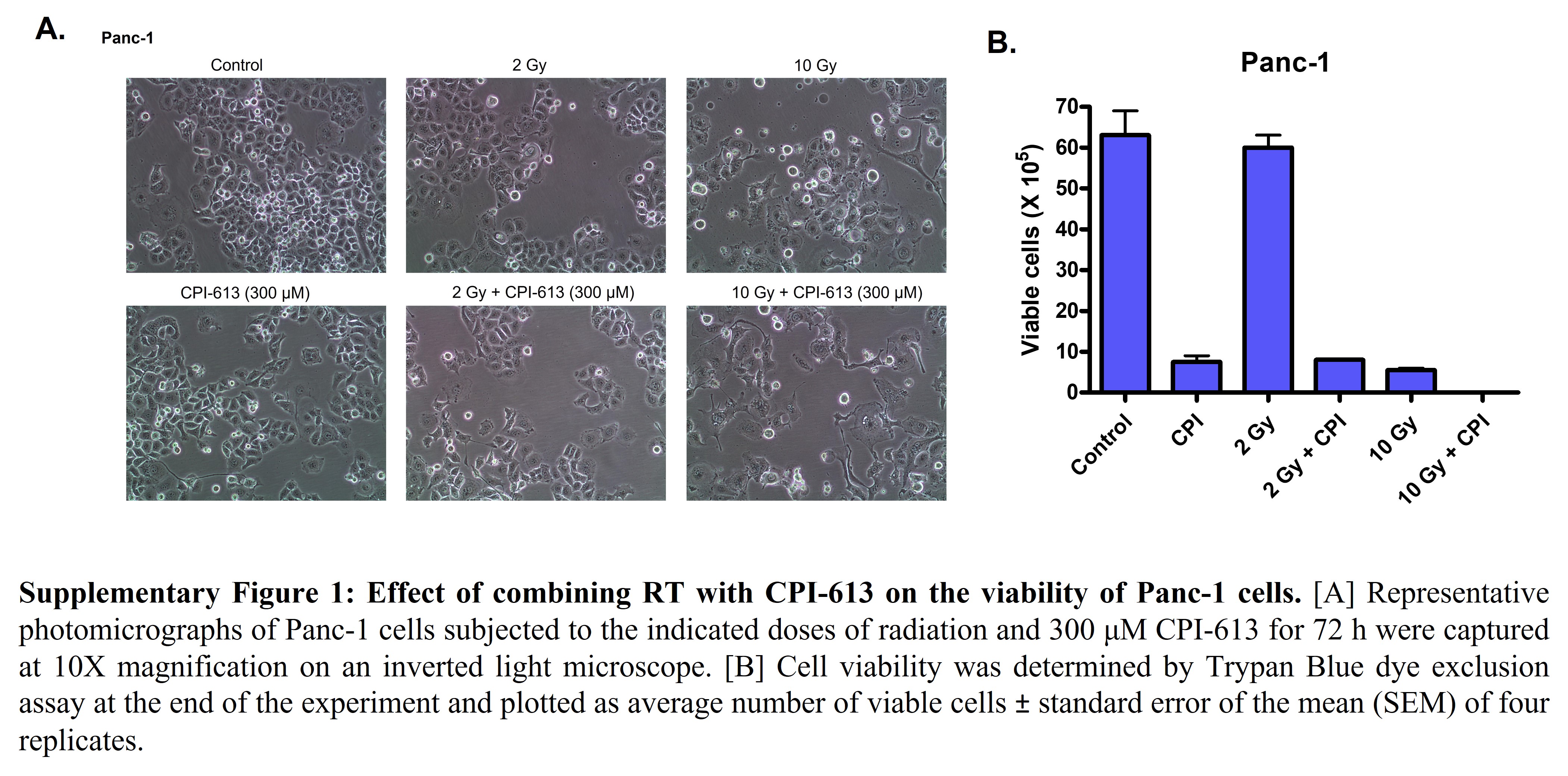

Supplement: Supplementary file 1 [file mmc1.jpg]
